# Off Earth Identification of Bacterial Populations Using 16S rDNA Nanopore Sequencing

**DOI:** 10.3390/genes11010076

**Published:** 2020-01-09

**Authors:** Aaron S. Burton, Sarah E. Stahl, Kristen K. John, Miten Jain, Sissel Juul, Daniel J. Turner, Eoghan D. Harrington, David Stoddart, Benedict Paten, Mark Akeson, Sarah L. Castro-Wallace

**Affiliations:** 1 Astromaterials Research and Exploration Science Division, NASA Johnson Space Center, Houston, TX 77058, USA; aaron.burton@nasa.gov; 2 JES Tech, Houston, TX 77058, USA; sarah.e.stahl@nasa.gov; 3 Jacobs Engineering Group Inc., Houston, TX 77058, USA; kristen.k.john@nasa.gov; 4 UCSC Genomics Institute, University of California, Santa Cruz, CA 95064, USA; miten@soe.ucsc.edu (M.J.); benedict@soe.ucsc.edu (B.P.); makeson@soe.ucsc.edu (M.A.); 5 Oxford Nanopore Technologies, New York, NY 10013, USA; Sissel.Juul@nanoporetech.com (S.J.); Eoghan.Harrington@nanoporetech.com (E.D.H.); 6 Oxford Nanopore Technologies, Oxford Science Park, Oxford OX4 4DQ, UK; Daniel.Turner@nanoporetech.com (D.J.T.); David.Stoddart@nanoporetech.com (D.S.); 7 Biomedical Research and Environmental Sciences Division, NASA Johnson Space Center, Houston, TX 77058, USA

**Keywords:** nanopore sequencing, in situ analysis, field-deployable methods, bacterial identification, spaceflight

## Abstract

The MinION sequencer has made in situ sequencing feasible in remote locations. Following our initial demonstration of its high performance off planet with Earth-prepared samples, we developed and tested an end-to-end, sample-to-sequencer process that could be conducted entirely aboard the International Space Station (ISS). Initial experiments demonstrated the process with a microbial mock community standard. The DNA was successfully amplified, primers were degraded, and libraries prepared and sequenced. The median percent identities for both datasets were 84%, as assessed from alignment of the mock community. The ability to correctly identify the organisms in the mock community standard was comparable for the sequencing data obtained in flight and on the ground. To validate the process on microbes collected from and cultured aboard the ISS, bacterial cells were selected from a NASA Environmental Health Systems Surface Sample Kit contact slide. The locations of bacterial colonies chosen for identification were labeled, and a small number of cells were directly added as input into the sequencing workflow. Prepared DNA was sequenced, and the data were downlinked to Earth. Return of the contact slide to the ground allowed for standard laboratory processing for bacterial identification. The identifications obtained aboard the ISS, *Staphylococcus hominis* and *Staphylococcus capitis*, matched those determined on the ground down to the species level. This marks the first ever identification of microbes entirely off Earth, and this validated process could be used for in-flight microbial identification, diagnosis of infectious disease in a crewmember, and as a research platform for investigators around the world.

## 1. Introduction

Technology to enable real-time crew health assessments, including monitoring the environment to which the crew is exposed, is key to the expansion of human space exploration. Characterization of the microbial environment of the International Space Station (ISS) has been ongoing since its inception [1]. Spaceflight crewmembers collect samples from the station’s air, water, and surfaces onto culture media to promote bacterial and fungal growth. Following an incubation period, the crew provides an approximation of colony forming units (CFU) to microbiologists at the Johnson Space Center (JSC), offering insight toward microbial load. The identity of the microorganisms is unknown until the samples are returned to Earth for laboratory analysis. With sights set toward the Moon and Mars, in which regular sample return will be impossible, there is a clear need for an in-flight microbial identification capability.

Sequencing is an incredibly powerful technique for microbial identification. However, nearly all commercially available sequencers are poorly suited for deployment in remote, low resource environments such as the ISS. The exception is the MinION (Oxford Nanopore Technologies) sequencer, which weighs less than 100 g and can have all of its power and data transfer provided through a single USB 3.0 connection. The MinION has been deployed in a range of field environments for infectious disease diagnosis [2,3], field microbial ecology [4,5,6], real time biodiversity assessments [7,8], and crop plant health [9]. Nanopore sequencing has also been shown to be an incredibly versatile platform, e.g., enabling whole genome sequencing and assemblies of fungi [10] and a human genome [11], as well as sequencing full-length RNA transcripts via both direct RNA and cDNA sequencing [12,13,14]. The MinION hardware and reagents have been shown to be radiation tolerant well beyond exposures that would be expected on a mission to the Moon or Mars [15]. This combination of portability and robustness make the MinION an attractive platform for sequencing aboard the ISS and on deep-space missions.

To determine whether sequencing could advance microbial monitoring and other areas of spaceflight research, ground-prepared samples were sequenced aboard the ISS [16]; these experiments demonstrated that there was no clear difference in sequencer performance on the ground versus the ISS. Additionally, in 2016, astronaut Dr. Kate Rubins demonstrated that pipetting with both positive displacement and air displacement pipettes could be achieved aboard the ISS [17], raising the possibility that a wide range of standard microbiology and molecular biology techniques could be performed in a very similar way to how they are performed in terrestrial laboratories. With these demonstrations in place, control DNA, reagents for sequencing library preparation, and flow cells were flown to the ISS for the Genes in Space-3 investigation to demonstrate that sample-to-sequencing entirely in-flight was possible.

In May of 2017, astronaut Dr. Peggy Whitson performed amplification of control DNA, clean-up reactions, library preparation, and sequencing in the spaceflight environment of the space station. Based on this success, the processing of a NASA Environmental Health Systems (EHS) microbial sample collected from and cultured aboard the ISS was initiated. Three bacterial colonies were selected from a surface sample culture slide. The cells were lysed and the 16S gene was amplified in a single step. After PCR clean-up, DNA libraries were prepared and sequenced. Sequence data were downlinked to Earth and the identifications documented. Upon receipt of the EHS culture slide at JSC, the same colonies were analyzed using standard laboratory procedures, confirming the space-derived identities. This marks the first identification of microbes entirely off Earth and paves the way for a new era of spaceflight science.

## 2. Materials and Methods 

### 2.1. Spaceflight Hardware 

The genes in Space-3 payload included several cold stowage items: R9.4 flow cells and reagents, including control DNA, Rapid 16S sequencing kits SQK-RAS201 (Oxford Nanopore Technologies (ONT), Oxford, UK), PCR master mix and exonuclease I (New England BioLabs (NEB), Ipswich, MA, USA), all aliquoted into spaceflight-certified tubes. A pipette kit including 20, 200, and 1000 μL Eppendorf Research Plus pipettes (Eppendorf Hamburg, Germany) with associated individually packaged tips was stowed at ambient temperature. The hardware was launched from Cape Canaveral Air Force Base on the Orbital ATK OA-7 Cygnus resupply vehicle on 18 April 2017. All reagents were launched and maintained at −90 °C, while flow cells were stowed and launched at + 4 °C. Upon docking with the ISS, the flow cells and reagents were transferred to the refrigerator (+2 to + 8 °C) and freezer (−80 to −90 °C) dewars within the Minus Eighty Degree Laboratory Freezer for ISS (MELFI), respectively.

### 2.2. Library Preparation and Sequencing (Ground and ISS) 

#### 2.2.1. 16S Amplification of DNA Standard 

Following removal from cold stowage, Hot Start Taq 2X Master Mix (NEB) containing 16S primers from the ONT 16S Rapid sequencing kit (SQK-RAS201) was pipetted into 10 ng of ZymoBIOMICS™ Microbial Community Standard (Zymo Research, Irvine, CA, USA) in three separate tubes of an 8 tube PCR strip. The reaction product was placed into a miniPCR (Amplyus, Cambridge, MA, USA) thermal cycler that was already onboard the ISS. PCR parameters were an initial denaturation of 95 °C for 1 min, followed by 30 cycles of 95 °C for 10 s, 60 °C for 10 s, 72 °C for 20 s, and a final extension of 72 °C for 90 s. All library preparations were completed in triplicate by pipetting with Eppendorf Research Plus pipettes on the open bench top. The amplified products were placed into the −80 to −90 °C MELFI dewar until they were used for library preparation. 

#### 2.2.2. Microbial Collection and Culture 

On 10 August 2017, the spaceflight crew of increment 53/54 collected a surface sample from a blackout curtain within the Permanent Multipurpose Module (PMM). To collect the sample, the crewmember touched a surface sample kit (SSK) tryptic soy agar (TSA) contact slide (Millipore Sigma, Burlington, MA, USA) to a portion of the blackout curtain. The slide was then placed into a Mylar incubation bag and stored at ambient ISS temperature, which can range from 18.3 to 26.7 °C [18], but is nominally maintained for crew comfort at approximately 22 °C, to allow for microbial growth. 

#### 2.2.3. Cell Lysis and 16S Amplification 

Within the microgravity science glovebox (MSG) [19] on 21 August 2017, Whitson opened the contact slide, which had bacterial and fungal growth, for the first time in spaceflight history. She selected three clearly defined bacterial colonies by characterizing the colonies, circling them with a Sharpie on the transparent case, and photographing the slide. Using a sterile, individually packaged, 50–1000 µL pipette tip (Eppendorf), she transferred a small portion of the colony to an individual PCR tube containing Hot Start Taq 2X Master Mix (NEB) and 16S primers from the SQK-RAS201 Rapid sequencing kit (ONT). This process was repeated twice more until all three colonies had been sampled. With the lids secured, the tubes were tapped to induce mixing. To ensure liquid collection at the bottom of the tubes and to force air bubbles to the surface, while holding the top of the tubes near the lid, Whitson firmly flicked her wrist to collect the contents at the bottom of the tube. This was repeated until the liquid was at the bottom of the tube and air bubbles had been eliminated or were only minimally present at the top of the air–liquid interface. The PCR tubes were then placed in the miniPCR device. The miniPCR was then removed from the MSG and connected to a Surface Pro3 and miniPCR v. 1.0 software. Lysis and amplification were achieved with the following thermal parameters: 95 °C for 5 min, 30 cycles of 95 °C for 10 s, 60 °C for 10 s, 72 °C for 20 s, and a final extension of 72 °C for 90 s. The amplified product was placed into a −80 °C freezer (NASA JSC) or the −80 to −90 °C MELFI dewar (ISS) until sequencing operations.

#### 2.2.4. Library Preparation and Nanopore Sequencing 

The action described above for the collection of liquid at the bottom of tubes was completed prior to the use of each reagent. Crew procedures dictated careful inspection of the tube and pipette tip for the presence of bubbles. If encountered, reagents were dispensed back into their tube and re-pipetted to reduce the transfer of bubbles. For both the amplified mock community standard and the ISS bacterial colony DNA, the PCR product was treated with Exonuclease I (NEB) and incubated at ambient temperature for 5 min. Ligation free rapid adaptor from SQK-RAS-201 diluted in a RAD dilution buffer (ONT) was then added to the reaction and incubated at ambient temperature for 5 min. An equal volume from each of the three tubes, containing either the amplified DNA standards or DNA amplified from ISS-derived organisms, were pooled into a tube containing flow cell loading buffer (ONT).

A R9.4 flow cell (ONT) was inserted in the MinION sequencer. Flow cell priming and loading was modified for optimal performance in microgravity. A 200 µL pipette was used to remove a volume of approximately 20 µL of air and flow cell storage buffer to reduce the risk of pushing an air bubble into the flow cell upon sample loading. Following flow cell priming with 200 µL of flush buffer (ONT) for approximately 10 min, 200 µL of the pooled DNA in loading buffer was injected into sample loading port. Sequencing was initiated through the offline MinKNOW software version 1.1.21 on the Surface Pro 3 using the NC_48hr_Lambda_ Exp_Flo-MIN106_SQK-RAD002.py protocol. Sequencing occurred for 48 h. Upon run completion, data were downlinked to the ground for analysis.

#### 2.2.5. Ground Processing and Identification of the SSK Contact Slide 

The contact slide from which the colonies were sequenced was returned to Earth on 3 September 2017, by the Soyuz 50 vehicle and returned to Houston, TX, on the crew plane. It was immediately transferred to the Microbiology Laboratory at NASA JSC, where it was logged and processed using routine procedures. Microbial colonies sequenced on orbit were clearly labeled, but high resolution photographs and video were used to confirm the markings. Using standard laboratory aseptic techniques, microbial colonies were streaked for isolation on TSA (Hardy Diagnostics, Santa Maria, CA, USA) and Gram stained. Biochemical assessments of the isolated bacteria were completed using Gram stain specific VITEK cards that were loaded and placed into the VITEK 2 Compact instrument according to the manufacturer’s directions (BioMerieux, Durham, NC, USA). DNA was extracted from isolated bacteria using the PrepMan™ ultra sample preparation reagent following the manufacturer’s protocols (ThermoFisher Scientific, Waltham, MA, USA) and identified by Sanger sequencing with MicroSeq 500 16S rDNA PCR and sequencing kits on an Applied Biosystems 3500 Genetic Analyzer (ThermoFisher Scientific). Isolated DNA was also sequenced on an Illumina MiSeq with a V2 cartridge (Illumina, San Diego, CA, USA). DNA was amplified and barcoded with the Earth Microbiome primers 515FB and 806R, as previously described [20,21,22,23,24]. Libraries were quantified using an Agilent 2100 bioanalyzer (Agilent Technologies, Santa Clara, CA, USA) and a Qubit spectrometer (ThermoFisher Scientific) and pooled for sequencing at an equal molar concentration following the manufacturer’s protocols for 16S metagenomic sequencing.

#### 2.2.6. Ground Controls 

While Whitson was preparing and sequencing ZymoBIOMICS Microbial Community Standard on the ISS, the payload team was replicating the procedure in the laboratory using duplicate reagents that were prepared at the same time as the flight reagents and stored at nearly identical temperatures for the same amount of time. For the ground control experiment that used ISS-derived organisms as input, the control experiment could not be performed until the contact slide had returned to Earth. The spaceflight procedure was repeated in the JSC laboratory with the isolated bacteria from the contact slide.

### 2.3. Data Analysis 

The reads were basecalled using Albacore (version 1.2.4) and all sequence alignments were performed using minimap2 (in -ax map-ont setting for nanopore reads). We used marginStats to calculate performance statistics [25]. For kraken classification we used a broad database consisting of complete bacterial, archaeal, and viral genomes. The VITEK 2 advanced expert systems (AES) software 8.01 assessed the organism’s minimum inhibitory concentration (MIC) patterns and detected phenotypes from various biochemical tests to determine species level identifications. Species level identifications were determined from Sanger sequencing using MicroSeq 2.0 database (ThermoFisher Scientific). MinION and Illumina reads are available at the European Nucleotide Archive (ENA) under accession number PRJEB35494. 

## 3. Results

**Figure 1:**
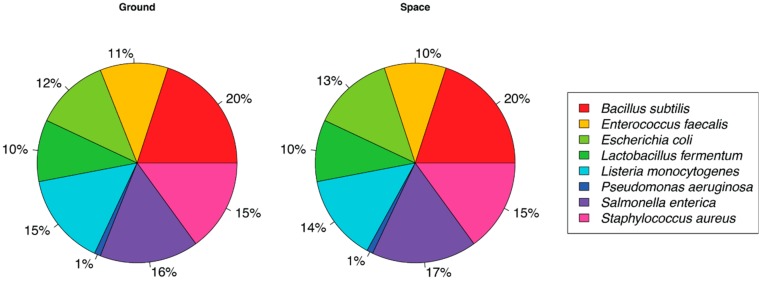
Percentage of reads assigned to ZymoBIOMICS Microbial Community DNA Standard reference genomes based on alignment using minimap2 (-ax map-ont). The space nanopore sequencing library was prepared and sequenced on the International Space Station by Whitson and compared to the ground control library prepared and sequenced at the Johnson Space Center Houston, TX using the same prepackaged reagent kits and protocols.

Whitson prepared a 16S nanopore sequencing library on the International Space Station using the ZymoBIOMICS Microbial Community DNA Standard (Zymo Research) while the science team simultaneously prepared a library using identical procedures and aliquots of reagents to those in-flight. The two sequencing datasets were basecalled using Albacore (version 1.2.4). We then aligned these sequence data to the microbial reference sequences provided by Zymo Research using minimap2 (with -ax map-ont setting for nanopore reads) and calculated alignment identity using marginStats15. Most of the reads were full length (Supplementary Figure S1). The median percent alignment identity for both the datasets (space and ground) was 84.3% (Supplementary Figure S2). The sequence identity in both datasets was comparable to what has been previously documented for nanopore sequencing using the same chemistry (R9.4, May 2017) [11]. We also calculated the proportion of reads assigned to respective species given the alignment and found them to be comparable between space and ground datasets (Figure 1) and to the microbial theoretical composition from Zymo Research. The ZymoBIOMICS Microbial Community Standard was also sequenced on an Illumina MiSeq, allowing a comparison of Illumina and nanopore data’s microbial classification using kraken [26] (Supplementary Table S1). We observed that long reads provided better classification at the genus level when searching against a broad database consisting of complete bacterial, archeal, and viral genomes. These results supported findings from Castro-Wallace et al. in 2017 that the spaceflight environment does not have a negative impact on nanopore sequencing quality and performance [16]. Both of the present runs had an average quality score of 10 and an average sequencing length of 1450 bases. This experiment went one step further to validate library preparation and pipetting on the orbit by comparing two libraries prepared using the exact same reagents and procedures simultaneously in space and on the ground. The crewmember noted no pipetting issues in the microgravity environment; surface tension was a driving factor in liquids staying in their respective tubes. 

**Figure 2:**
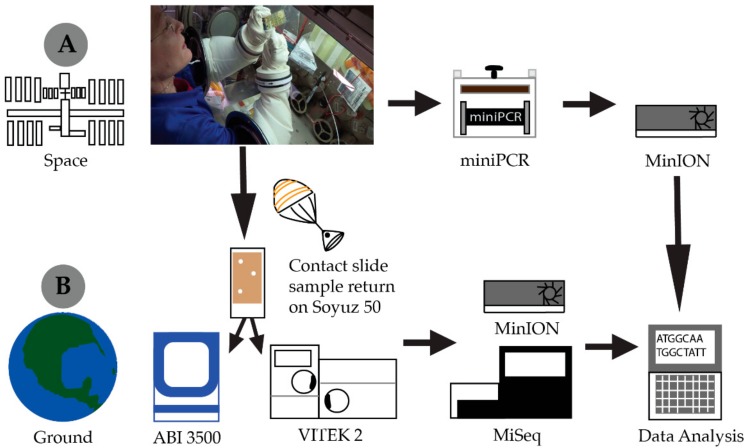
Workflow of the first on orbit sequencing library preparation and in situ sequencing of bacterial colonies cultured from the International Space Station performed by astronaut Dr. Peggy Whitson on 21 August 2017. (**A**). Whitson selected three bacterial colonies from a surface sample kit slide for colony PCR amplification. Amplified DNA was cleaned up with Exonuclease 1 enzyme and prepared for sequencing with Rapid 16S sequencing kit (RAS201 ONT). After 48 h of sequencing, data were downlinked to Earth for analysis. (**B**). The bacterial culture was returned to Earth on Soyuz 50 and transported to the Johnson Space Center Microbiology Lab for routine biochemical and Sanger sequencing analysis, as well as additional Nanopore and MiSeq sequencing.

**Figure 3:**
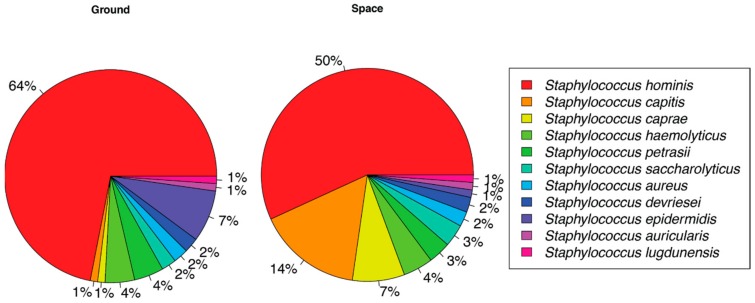
Percentage of reads assigned to microbial species cultured on the International Space Station and analyzed through in situ nanopore sequencing and return analysis on Earth. Reads were assigned to species in the National Center for Biotechnology Information 16S database based on alignments using minimap2 (-ax map-ont). The microbial culture was performed aboard the International Space Station. Nanopore sequencing library was prepared and sequenced on the International Space Station by Whitson. Ground control library was prepared and sequenced at the Johnson Space Center Houston, TX using the same prepackaged reagent kits.

This validation of library preparation in-flight led to the first ever identification of unknown environmental microorganisms cultured aboard the ISS (Figure 2). As part of the standard environmental monitoring process, crewmembers use agar contact slides to collect and culture microbes from the habitable volume of the ISS. These samples are incubated for five days at ambient temperature and then assigned an approximate colony count, based on comparison to a density chart, which is reported to the Mission Control Center (MCC) in Houston, TX. Identification is not possible until samples are returned to the laboratory. Out of consideration for the safety of the crew, the culture plates are not opened aboard the ISS. As such, this study marked the first occasion of opening a microbial culture slide aboard the ISS. Based on routine monitoring data, it was expected that the microorganisms cultured would be Biosafety Level-1 (BSL-1), but the presence of BSL-2 organisms could not be ruled out. Consequently, opening of the plate and collection of the cells was completed within the microgravity science glovebox (MSG) to ensure containment of the sample. The slide selected for downstream processing was from a curtain providing privacy to the permanent multipurpose module (PMM). Based on colony morphology, Whitson selected three individual bacterial colonies for the identification process (Figure 2). This slide, along with other air and surface samples, was returned to the JSC on 3 September 2017. With the colonies selected for in-flight analysis clearly marked, they were subcultured as per standard operating procedures. Ground-based analyses included biochemical assessments, Sanger, Illumina, and nanopore sequencing. The nanopore data generated from microbial samples cultured aboard the ISS and returned to the ground were analyzed using parallel strategies. First, we used the 16S workflow available to MinION users as a standard tool via ONT’s EPI2ME platform (Supplementary Tables S2 and S3). We also performed an alignment of these reads to the National Center for Biotechnology Information (NCBI) reference database for 16S sequences using minimap2 (with -ax map-ont setting), as shown in Figure 3. These two analysis platforms showed a similar species level sample identification. Nanopore sequencing of the ISS cultures identified *Staphylococcus hominis* and *Staphylococcus capitis* as the most abundant microbial species. The ground culture had *S. hominis* and *Staphylococcus epidermis* as the most abundant microbes. Illumina Miseq of the three returned isolates identified *Staphylococcus* as the predominant genus present in the sample but a species level identification was not possible (Supplementary Table S4). Culture-based analysis revealed that all three isolates were Gram-positive cocci with biochemical traces matching *S. hominis*, *S. hominis,* and *S. capitis* at 97%, 97%, and 94% identification score, respectively (Table 1). Sanger sequencing of DNA extracted from cultured plates corroborated the VITEK results with identification matches at 99–100% accuracy (Table 1). 

The three colonies selected for sequencing in-flight were not the only isolates present on the plate at the time and additional colonies had grown on the plate during the return process. The returned plates had also been packed very tightly leading to some mixing of organisms on the plate. Microbiologists in the laboratory also identified *S. aureus* and *S. epidermidis* (outside of the areas marked by Whitson) in addition to *S. hominis* and *S. capitis* on the plate. The observation that the ground nanopore sequencing results performed on the post flight samples do not perfectly match the identifications obtained from in-flight sequencing further demonstrates the value of in-flight sequencing-based microbial monitoring over the current return-based procedures where samples can often become contaminated or overgrown, complicating organism identification. 

## 4. Discussion

As the Earth’s only perpetual microgravity environment, the ISS affords unique opportunities to study the effects of long-term microgravity on biological systems. To date, several adverse effects on humans have been identified, including bone loss and immune system dysregulation [27,28]. Among microbial populations, increased virulence has been observed in some cases as a response to spaceflight [29]. However, the molecular basis for these changes have only begun to be elucidated. This is in part because capabilities such as in-flight sequencing were not available, and experiments have been forced to rely on the periodic return of frozen samples or organisms and their immediate analysis upon return to Earth. This return process poses many logistical challenges including time from sample collection to receipt, sample storage, and exposure to very different conditions during Earth return than were experienced on the ISS.

In conclusion, this sample-to-sequencing capability has been demonstrated entirely in-flight aboard the ISS. This powerful and versatile platform would enable researchers and medical operations personnel to collect and transmit data to Earth in a matter of hours, rather than waiting weeks or months for samples to be returned to Earth. This improvement in speed would directly benefit crew health by permitting rapid identification of microbes, including for infectious disease diagnosis. Researchers would also benefit by being able to analyze some of their data during the course of their experiments, enabling previously unavailable opportunities to make changes to the experiments such as adjusting time-points or changing how procedures are implemented.

## Figures and Tables

**Table 1:** Sample identification of returned microbial samples from ISS using VITEK 2 biochemical profiling and 3500 Sanger Sequencing.

Colony	Detection Method	Sample ID	%ID
1	Biochemical	*Staphylococcus hominis hominis*	97.0
2	Biochemical	*Staphylococcus hominis hominis*	97.0
3	Biochemical	*Staphylococcus capitis*	94.0
1	Sanger Sequencing	*Staphylococcus hominis hominis (ATCC = 27,844)*	99.9
2	Sanger Sequencing	*Staphylococcus hominis hominis (ATCC = 27,844)*	100.0
3	Sanger Sequencing	*Staphylococcus capitis capitis (ATCC = 27,840)*	99.9

## Supplementary Materials

The following are available online at https://www.mdpi.com/2073-4425/11/1/76/s1. Figure S1: Read length histograms for nanopore data from ground and space sequencing of ZymoBIOMICS Microbial Community Standard; Figure S2: Alignment identity vs. read length for nanopore data from ground and space sequencing of ZymoBIOMICS Microbial Community Standard; Table S1: Summary of Kraken output at the genus level for ZymoBIOMICS Microbial Community Standard; Table S2: Top 12 species level identifications from nanopore sequencing of ISS data from Epi2ME; Table S3: Top 12 species level identifications from nanopore sequencing of ground data from Epi2ME; Table S4: Summary of Kraken output at the genus level for bacteria cultured aboard ISS.
