# Supplementary material for: Off Earth Identification of Bacterial Populations Using 16S rDNA Nanopore Sequencing
Source: Genes (Basel). 2020 Jan 9;11(1):76. doi: 10.3390/genes11010076 (PMC7016637; doi:10.3390/genes11010076)
Supplement: Supplementary file 1 [file genes-11-00076-s001.pdf]

Ground	Space
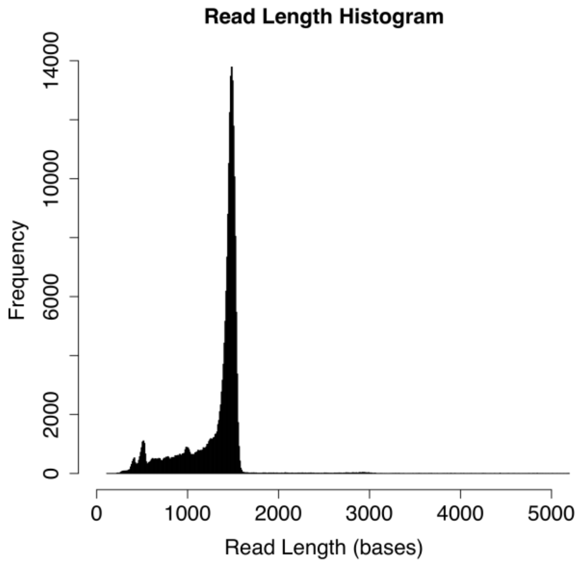	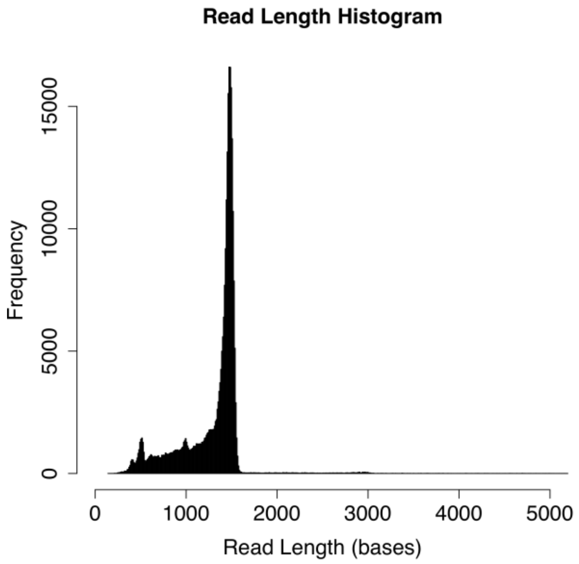

Supplementary Figure 1. Read length histograms for nanopore data from ground and space sequencing of ZymoBIOMICS Microbial Community Standard.

Ground	Space

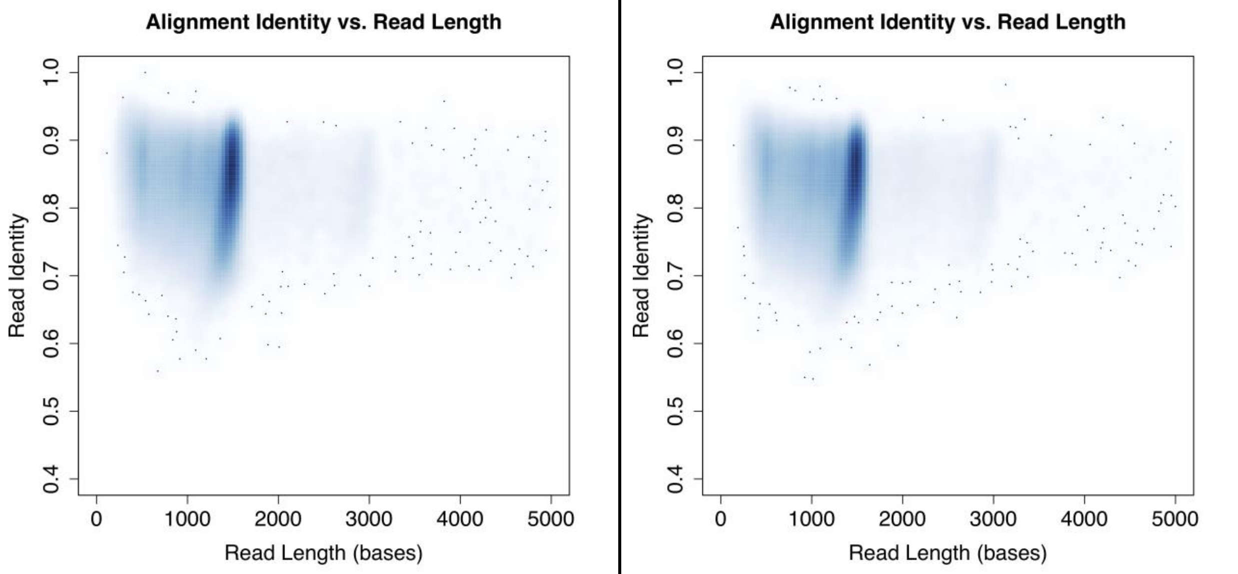

Supplementary Figure 2. Alignment identity vs. read length for nanopore data from ground and space sequencing of ZymoBIOMICS Microbial Community Standard.

Supplementary Table 1. Summary of Kraken output at the genus level for the ZymoBIOMICS Microbial Community Standard

	Proportion (%)		
**Genus**	**MiSeq (Ground)**	**Nanopore (Ground)**	**Nanopore (Space)**
Bacillus	16.97	18.05	17.71
Enterococcus	0.37	5.38	5.01
Escherichia	1.13	9.86	11.23
Lactobacillus	13.64	8.55	8.42
Listeria	13.59	8.86	8.28
Pseudomonas	5.82	0.9	0.63
Salmonella	1.82	4.8	5.38
Staphylococcus	17.9	11.95	11.45
Unclassified*	0.03	15.47	14.47

*The majority of unclassified reads from the nanopore data were either significantly shorter (<1kB) or longer (>2kB) than the expected 16S amplicon size, and are most likely due to spurious PCR products or ionic current aberrations.

Supplementary Table 2. Top 12 Species level identifications from nanopore sequencing flight data from EPI2ME.

**Taxon**	**Cumulative Reads**
Staphylococcus hominis	14,674
Staphylococcus capitis	4,031
Staphylococcus caprae	1,440
Staphylococcus saccharolyticus	862
Staphylococcus petrasii	859
Staphylococcus haemolyticus	233
Staphylococcus aureus	195
Staphylococcus epidermidis	173
Staphylococcus devriesei	165
Staphylococcus saprophyticus	88
Staphylococcus lugdunensis	77
Staphylococcus cohnii	75

Supplementary Table 3. Top 12 species level identification of nanopore sequencing ground data from EPI2ME.

**Taxon**	**Cumulative Reads**
Staphylococcus hominis	109,788
Staphylococcus epidermidis	9,107
Staphylococcus petrasii	5,635
Staphylococcus saccharolyticus	3,958
Staphylococcus haemolyticus	1,666
Staphylococcus capitis	1,169
Staphylococcus devriesei	1,112
Staphylococcus saprophyticus	707
Staphylococcus caprae	544
Staphylococcus aureus	536
Staphylococcus lugdunensis	526
Staphylococcus muscae	526

Supplementary Table 4. Summary of Kraken output at the genus level for bacteria cultured aboard ISS.

	Proportion (%)		
**Genus**	**MiSeq (Ground)**	**Nanopore (Ground)**	**Nanopore (Space)**
Staphylococcus	98.06	66.3	59.61
Bacillus	0.06	4.95	5.03
*Unclassified**	0.02	19	24.53

*The majority of unclassified reads from the nanopore data were either significantly shorter (<1kB) or longer (>2kB) than the expected 16S amplicon size, and are most likely due to spurious PCR products or ionic current aberrations.
